# Feature-Based Characterisation of Turned Surface Topography with Suppression of High-Frequency Measurement Errors

**DOI:** 10.3390/s22249622

**Published:** 2022-12-08

**Authors:** Przemysław Podulka

**Affiliations:** Faculty of Mechanical Engineering and Aeronautics, Rzeszow University of Technology, Powstancow Warszawy 8 Street, 35-959 Rzeszów, Poland; p.podulka@prz.edu.pl; Tel.: +48-17-743-2537

**Keywords:** surface topography, surface measurement, measurement noise, measurement errors, feature characterisation

## Abstract

Errors that occur when surface topography is measured and analysed can be classified depending on the type of surface studied. Many types of surface topographies are considered when frequency-based errors are studied. However, turned surface topography is not comprehensively studied when data processing errors caused by false estimation (definition and suppression) of selected surface features (form or noise) are analysed. In the present work, the effects of the application of various methods (regular Gaussian regression, robust Gaussian regression, and spline and fast Fourier Transform filters) for the suppression of high-frequency measurement noise from the raw measured data of turned surface topography are presented and compared. The influence and usage of commonly used available commercial software, e.g., autocorrelation function, power spectral density, and texture direction, which function on the values of areal surface topography parameters from selected (ISO 25178) standards, are also introduced. Analysed surfaces were measured with a stylus or via non-contact (optical–white light interferometry) methods. It was found that the characterisation of surface topography, based on the analysis of selected features, can be crucial in reducing measurement and data analysis errors when various filters are applied. Moreover, the application of common functions can be advantageous when feature-based studies are proposed for both profile and areal data processing.

## 1. Introduction

Concerning all studies assigned to surface science and surface engineering, the processes of measuring and analysing the data of surface topography plays an even more significant role than precision in manufacturing and requires a comprehensive approach. From that matter, even accurate measurement processes, including highly precise measurement equipment, may not allow for obtaining relevant results. Usually, when considering the measurement process, both the measuring device and parameters are presented, but so far, the data analysis processes have yet to be introduced properly.

Development is surface roughness measurement techniques focuses on the reduction in time and, respectively, it can be received when using stylus techniques compared to those optical [1]. However, when non-stylus methods are used, the probability of selected measurement errors may increase [2]. All surface topography measurement errors can be roughly divided into typical errors concerning the measuring method [3], those caused by the digitisation or data processing [4], and further, software [5], measuring object [6], or other [7,8] errors.

One of the errors that occurs when the measurement process is provided is measurement noise [9]. There are many types of measurement noise when measuring surface topography [10], such as instrument or white [11], random [12], phase [13], and signal-to-ratio [14] noises. However, the measurement noise in the high-frequency domain [15] is studied in the present analysis. Generally, in the non-contact (optical) measurement of surface (topography), according to the non-uniform distribution of the light intensity, the received signal is susceptible to being skewed and asymmetric; therefore, a lot of high-frequency noise can be included [16]. Seeking the source, high-frequency noise can be caused by the mechanics’ instability, considering any influences from the environment, but, in most cases, this type of error is the result of vibration [17,18].

For the extraction of relevant features and properties from the raw measured data, many sophisticated algorithms and procedures have been proposed. Moreover, filters are very popular in surface metrology. A wide range of digital filters were reviewed in refs. [19,20,21]. They are often represented by a Gaussian filter [22,23] and plenty of its modifications [24,25,26]. Spline filters are also very popular in the measurement and analysis of surface topography [27,28], e.g., two-dimensional isotropic spline filters [29], cubic spline filters [30], B-spline filtering techniques [31], or other combinations [32,33]. Another frequency-based technique is spectral analysis, widely introduced as a fast Fourier transform (FFT) method [34]. It was found that the fast Fourier transform filter (FFTF) can be valuable in both profile (2D) and areal (3D) studies [35] when considering surface topography measurements [36]. Reviewing further, wavelets are very common [37], resolving many metrological issues [38,39]. It was found that the application of wavelets can be classified as a manufacturing signature [40]. Furthermore, the use of morphological filters [41,42] with open and closing operations can also be very valuable [43,44,45].

As seen from previous studies, there are many algorithms, procedures, and approaches concerning the measurement of surface metrology performance. However, guidance on how to deal with many measurement issues must be precise, and valuable information is still urgently required. Application of regular methods, such as those available in commercial measuring software, can be fraught with many errors in method selection that affect the validation of the manufactured parts while control is performed. It was found in the previous studies of the author that concerning the effect of surface topography filtering (calculation of ISO 25178 parameters in the surface roughness evaluation), feature size, density, and distribution have a significant influence when using regular common methods.

For various surface topographies, many emerging technologies of feature-based studies have been introduced to achieve a more direct relationship between characterisation, manufacturing process, and surface function. Feature-based schemes contribute to the dimensional analysis of individual features, e.g., area, width, and height. Furthermore, the statistical properties of feature aggregations, such as mean or standard deviation, may be more intuitive or related to functionality and could be assessed with a feature-based characterisation. Moreover, isolating relevant topographic formations, regularly defined as features, is a developing field of study in surface topography measurement [46,47]. Examples of feature characterisation are methods with excluding valleys approaches [48,49].

In this paper, the influence of commonly used (available in commercial software) algorithms (filters) was provided with the application of selected feature-based techniques. It was proposed to use an analysis of some features to validate selected procedures for detecting and reducing some frequency-based errors, such as high-frequency noise. In previous studies, the feature-based decomposition approach (FBDA) was improved, and direction methods were valuable in the characterisation of turned topographies [50] or topographies with a defined direction [51]. The frequency analysis of surface topography can also be valuable in the determination of similarities [52] with the application of 2D FFT, where the description of the lateral resolution criterion for profile and areal surface topography measuring instruments is required according to the discussion in the international standardisation. Comprehensive examination and comparison of the different methods proposed by the standard were presented by ref. [53]. It was determined that the selection of features for improving data analysis procedures could be especially valuable when calculating areal (3D) surface topography parameters from ISO standards.

## 2. Materials and Methods

### 2.1. Analysed Surfaces

**Figure 1:**
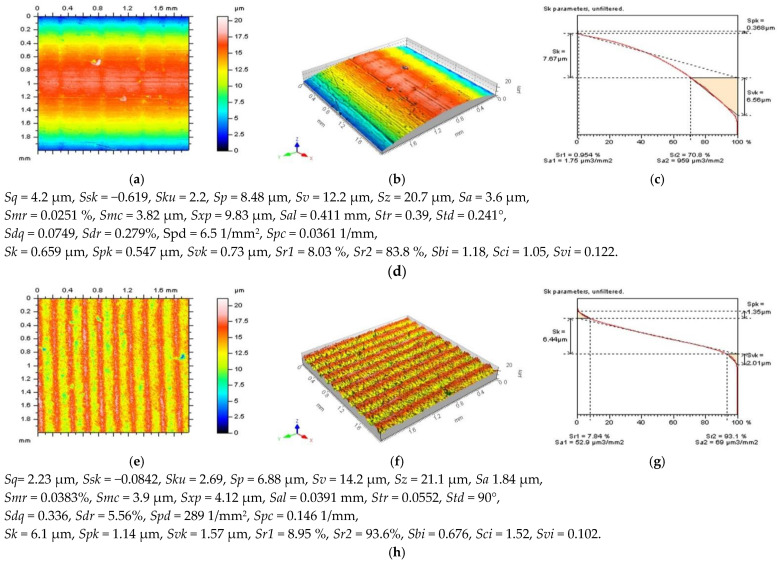
Contour map plots (**a**,**e**), isometric views (**b**,**f**), material ratio curves (**c**,**g**), and ISO 25-178 parameters (**d**,**h**) of turned surface: measured data (**a**–**d**) and data after an areal form removal by application of a least square fitted polynomial of 8th degree (**e**–**h**).

Various surface types were studies after the turning process. In Figure 1, two examples of turned surfaces topographies and their parameters are presented: amplitude (root-mean-square height Sq, skewness Ssk, kurtosis Sku, maximum peak height Sp, maximum valley depth Sv, the maximum height of surface Sz, arithmetic mean height Sa), functional (areal material ratio Smr, inverse areal material ratio Smc, extreme peak height Sxp), spatial (auto-correlation length Sal, texture parameter Str, texture direction Std), hybrid (root-mean-square gradient Sdq, developed interfacial areal ratio Sdr), feature (peak density Spd, arithmetic mean peak curvature Spc), functional indices (surface bearing index Sbi, core fluid retention index Sci, valley fluid retention index Svi), and ISO 25178 from the SK family (core roughness depth Sk, reduced summit height Spk, reduced valley depth Svk, upper bearing area Sr1, lower bearing area Sr2). The surface in the first row (Figure 1a,b) is raw measured data, and the surface from the third row (Figure 1e,f) was obtained after areal form removal by application of a polynomial of the 8th degree [54].

More than 20 turned surfaces were measured and studied, but only selected ones were presented in detail. For direct validation of the procedures proposed, more than 20 surfaces with modelled data (high-frequency measurement errors) were considered.

### 2.2. Measurement Process

Analysed turned surfaces were measured by various (stylus or optical) methods to provide proposals for different measurement techniques.

The Talyscan 150 stylus instrument was equipped with a nominal tip radius of approximately 2 μm, a height resolution of 10 nm, a measured area of 5 by 5 mm with 1000 × 1000 measured points, and a sampling interval of 5 μm. The measurement speed was 1 mm/s. Correspondingly, its influence was not considered in this paper; the issue was comprehensively studied previously.

The white light interferometer Talysurf CCI Lite (produced by Taylor Hobson Ltd., Leicester, UK, version 2.8.2.95), a non-contact measurement device, was also employed with a height resolution of 0.01 nm, a measured area of 3.35 by 3.35 mm with 1024 × 1024 measured points, and spacing of 3.27 μm. A Nikon 5×/0.13 TI objective was used for all the optical measurements. The effect of sampling on areal texture parameters was not studied in this paper.

For both analyses, TalyMap Gold software, copyright by Digital Surf, was used for digital filtering (regular Gaussian regression, robust Gaussian regression, spline, and fast Fourier transform filters) and data analysis (application of the autocorrelation function, power spectral density, and texture direction).

### 2.3. Applied Methods

Turned surface topographies were studied to reduce the influence of high-frequency measurement noise on the results obtained, including the ISO 25178 parameters. Generally, the reduction of the influence of measurement errors can be roughly divided into two separate processes. First, the measurement noise must be detected (defined), and then the appropriate method can be applied to remove irrelevant components from the raw (measured) data.

To improve the proposed procedures, general, commonly used, and available commercial software, algorithms, and procedures, such as (for high-frequency noise detection) power spectral density (PSD), autocorrelation function (ACF), and texture direction (TD), or (for high-frequency measurement noise reduction), a regular Gaussian (regression) filter (GF), robust Gaussian (regression) filter (RGF), regular isotropic spline filter (SF), or fast Fourier transform filter (FFTF), were proposed.

It was found in previous studies of the author that PSD graphs can be extremely valuable in the detection of high-frequency measurement errors. PSD, in its two-dimensional form, has been designated on the draft international drawing standard for surface texture analysis. However, the proposal of a method for the variance of PSD estimate reduction must also be defined [55]. Moreover, PSD was proposed on topography data from a range of optical surfaces when varying quality and manufacturing techniques. This can be especially valuable when a direct comparison of metrology data is received using instruments with different spatial bandwidths [55].

Another function available in commercial software is ACF. When analysing the ACF, it provides practical advice regarding the autocorrelation length and its properties as a function of surface irregularities [56]. When comparing PSD and ACF functions, ACF is more accurate for the study of irregular surfaces, but PSD is better for the analysis of periodic surfaces [57]. As another advantage, the ACF method can be applied for the generation of rough surfaces when using both the 2D digital filter and the Fourier analysis method but with controlled ACF and height distribution [58]. 

The character of the surface, especially its isotropic or anisotropic properties or directionality, can be provided with an angular spectrum [59]. An anisotropy, which is crucial when indicating the influence of surface function and processing many surface topography properties, can be received when multi-scale studies are proposed [60,61]. Therefore, the TD graph can be used when a high-frequency noise is studied in the characterisation of surface topography. 

The removal (reduction) of high-frequency measurement errors can be provided with regular filters, also available in commercial software. Firstly, the GF, which is very popular in the analysis of surface topography, has been widely presented in many standards (e.g., firstly in ISO 11562:1996 [62] and revised in ISO 16610-21:2011 [23]) when evaluating surface roughness. However, when the surface contains some extraordinary results (usually points, e.g., spikes), using the GF, it is difficult to extract the shape components [63]. To resolve this problem, a robust modification of the GF was proposed, receiving the RGF [26]. Splines are often applied in surface roughness evaluation [64], presenting many advantages against regular Gaussian filtering methods [65]. For the separation of the roughness, waviness, and form components of the surface topography, the regular isotropic spline filter can be proposed. The fractional spline filter, often defined as the universal spline filter [66], is calculated by a fast Fourier transform. Fast Fourier filtration, FFTF, of the surface topography has been proposed in many recently published studies [67,68,69,70,71,72,73].

**Figure 2:**
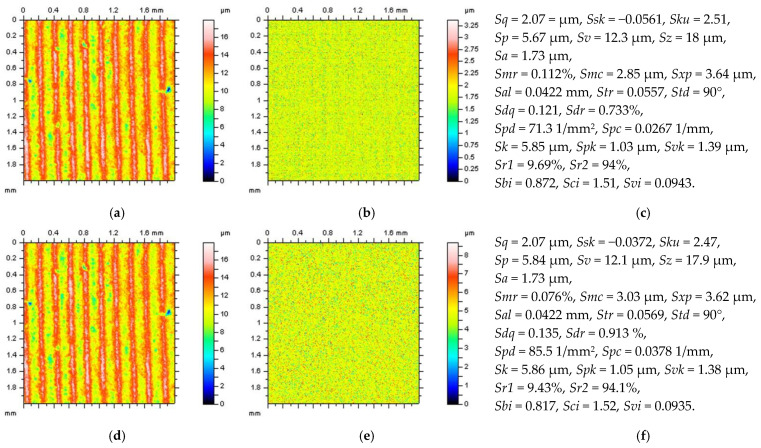

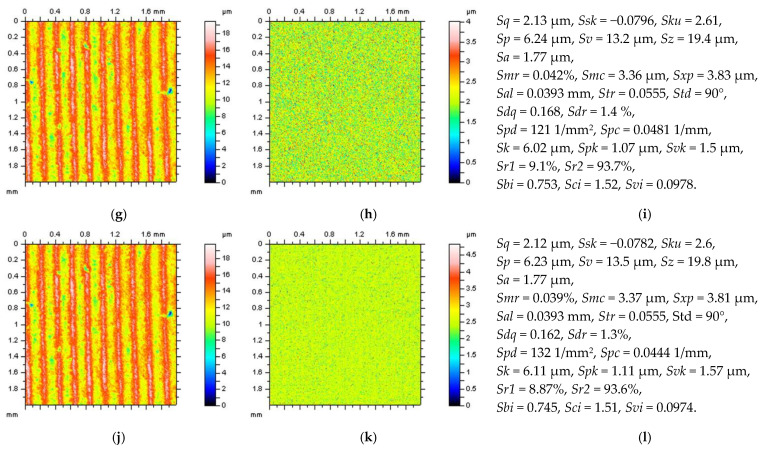
The turned surface after noise removal: contour map plots of the data with removed noise (**a**,**d**,**g**,**j**), contour maps plots of NSs (**b**,**e**,**h**,**k**), and the ISO 25178 topography parameters of the surface after noise reduction (**c**,**f**,**i**,**l**), respectively, received after application of GF (**a**–**c**), RGF (**d**–**f**), SF (**g**–**i**), and FFTF (**j**–**l**). Cut-off = 0.015 mm.

**Figure 3:**
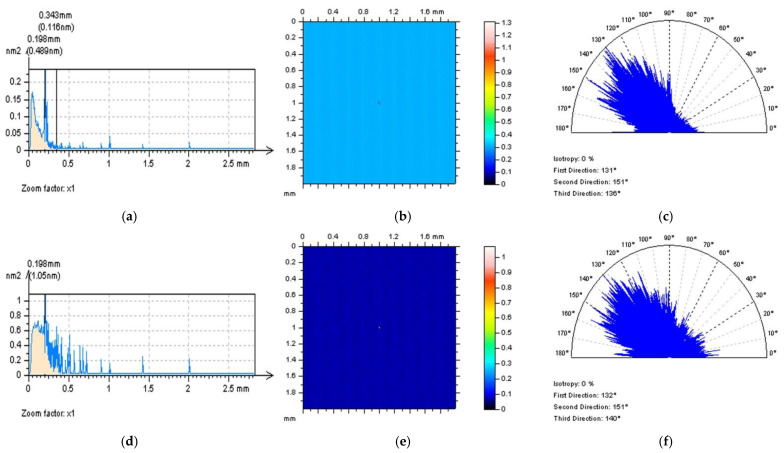

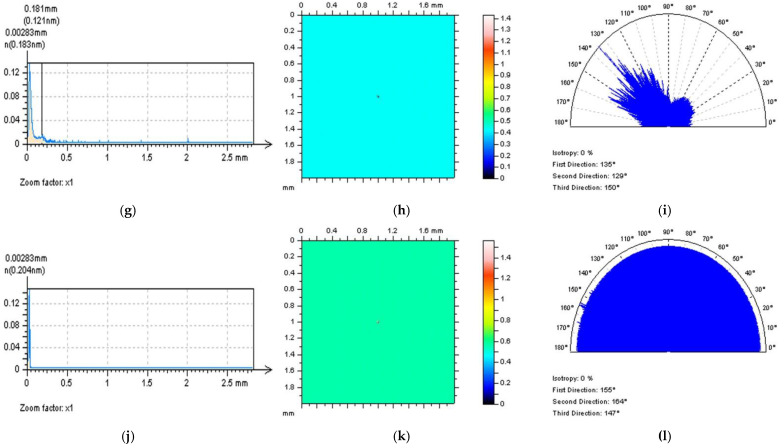
PSDs (left column), ACFs (middle), and TDs (right column) of NSs received from the turned surface topography after application of GF (**a**–**c**), RGF (**d**–**f**), SF (**g**–**i**), and FFTF (**j**–**l**). Cut-off = 0.015 mm.

All of the proposed methods for high-frequency measurement error detection (definition) and reduction were compared and presented for turned surface analysis. It was found that the results that were removed from a primary (raw measured data) surface when the denoising (reduction of high-frequency noise) process was applied were defined as a noise surface (NS). The properties of an NS were studied comprehensively previously [27]. In Figure 2, various NSs are presented after the application of different filtering methods. By observing the PSD and TD graphs (Figure 3), it can be indicated that for analysed turned surfaces, FFTF provides the best results (from presented, regular filters) in high-frequency measurement noise extraction.

All of the presented studies consider the reduction of the effect of high-frequency measurement noise on the surface topography assessments, including ISO 25178 parameter analysis. Raw measured data were first processed for areal form removal. The selection of the method for form and waviness elimination was widely studied previously [4,5,74], so was not considered in this research.

The main purpose of this study was to divide the received data (surface) into the surfaces containing different features, such as valleys and dimples, and, correspondingly, indicate the influence of feature occurrence on the process of high-frequency measurement noise reduction (detection and removal). The effect of both density and location of the features, e.g., oil pockets, dimples, valleys, or scratches, in the analysed surface was not introduced as it has already been comprehensively studied.

For validation of the selection process, the noise surface (NS) was characterised using ACF, PSD, and TD approaches. The method for high-frequency measurement error reduction was proposed depending on the features located in the surfaces analysed. The main purpose was not to propose one approach (e.g., digital filters such as GF, RGF, SF, or FFTF) but to present procedures on how to select appropriate algorithms for high-frequency measurement noise reduction with the usage of a method available in commercial software. From that point, the user, when applying various digital filters and functions, should select the appropriate procedure depending on the suitable properties of the S-surface, defined in this paper as NS.

**Figure 4:**
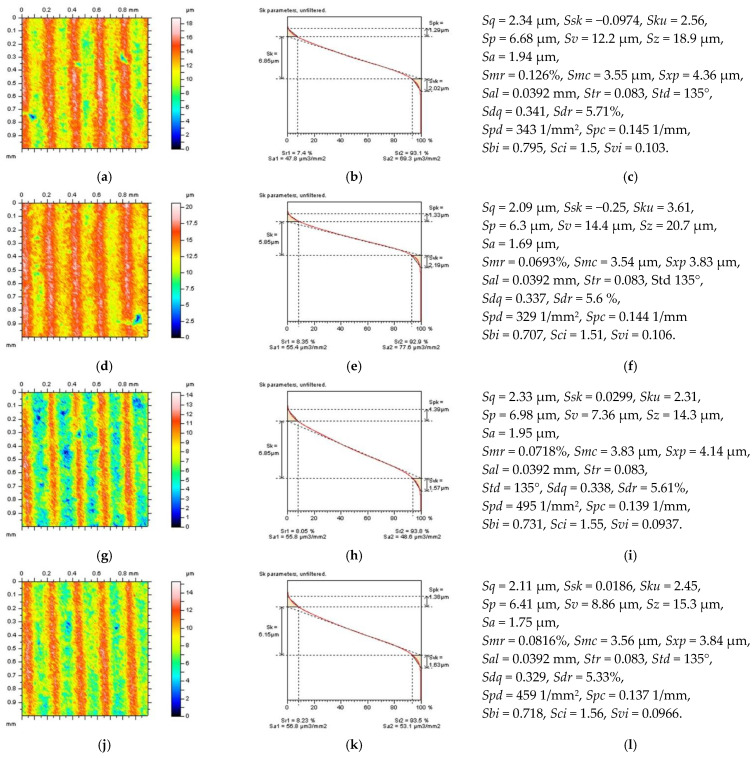
Contour map plots (left column), material ratio curves (middle), and ISO 25178 topography parameters (right column) of the turned surface received from the surface after a feature-based dividing process: surface A with one valley feature and one pit feature (**a**–**c**), surface B with one valley feature (**d**–**f**), surface C with one pit feature (**g**–**i**), and surface D not containing any extraordinary features (**j**–**l**).

One of the proposed techniques was to divide the surface into areas containing a selected number of features (Figure 4). From that matter, surface A contains two features, surfaces B and C one feature, and surface D does not have features. Except for the location of the feature, their densities can radically influence the process of high-frequency measurement noise detection. In the considered case, this was not studied as some sophisticated analysis was provided previously, e.g., for plateau-honed cylinder liners containing oil pockets. Not only are these features recognizable by studies of the isometric view of the surface, but the differences can be observed for ISO 25178 (especially Sv, Sz, and Spd) and Sk group (Spk and Svk) parameters as well.

**Figure 5:**
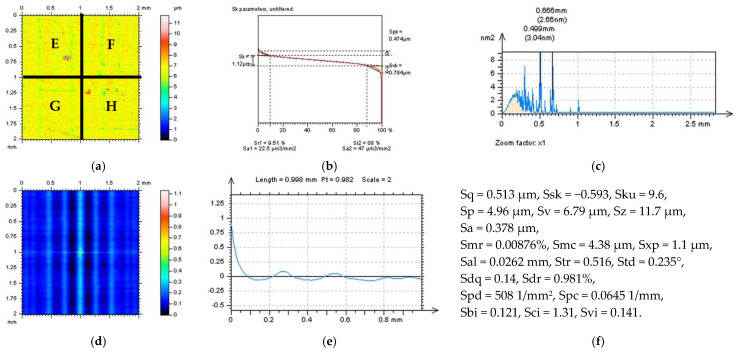
Contour map plot (**a**), material ratio curves (**b**), PSD (**c**), 3D (**d**), and 2D (**e**) ACF with ISO 25178 topography parameters (**f**) received from the turned surface containing several features.

The presentation and discussion of the results obtained are divided into two crucial sections. Firstly, in Section 3.1, regular, available commercial software methods are compared with the application of a feature-based technique. Secondly, in Section 3.2, improvement in the selection of a digital filtering cut-off value is presented with the application of feature-based characterisation. In Figure 5, the process of dividing the measured turned surface into four separate (E, F, G, and H) surfaces is presented. Each of them contains different features with various densities, analysed further in Section 3.1. and 3.2.

## 3. Results and Discussion

### 3.1. Comparison of Regular and Feature-Based Methods for a High-Frequency Noise Detection

When defining a proper method to reduce the influence of high-frequency measurement errors, it is suggested to use a multithreaded analysis [27]. This method includes studies of the isometric view of the NS, its PSD, ACF, and TD, respectively. When considering the properties of NS, it should only be included with high-frequency components and should be isotropic [57]. Moreover, the NS should not contain any (other than high frequency) features, such as dimples, scratches, holes, pits, etc. For that matter, the shortest way to analyse NSs, if received by the selected methods (digital filtering), is to study the isometric view of the NS.

**Figure 6:**
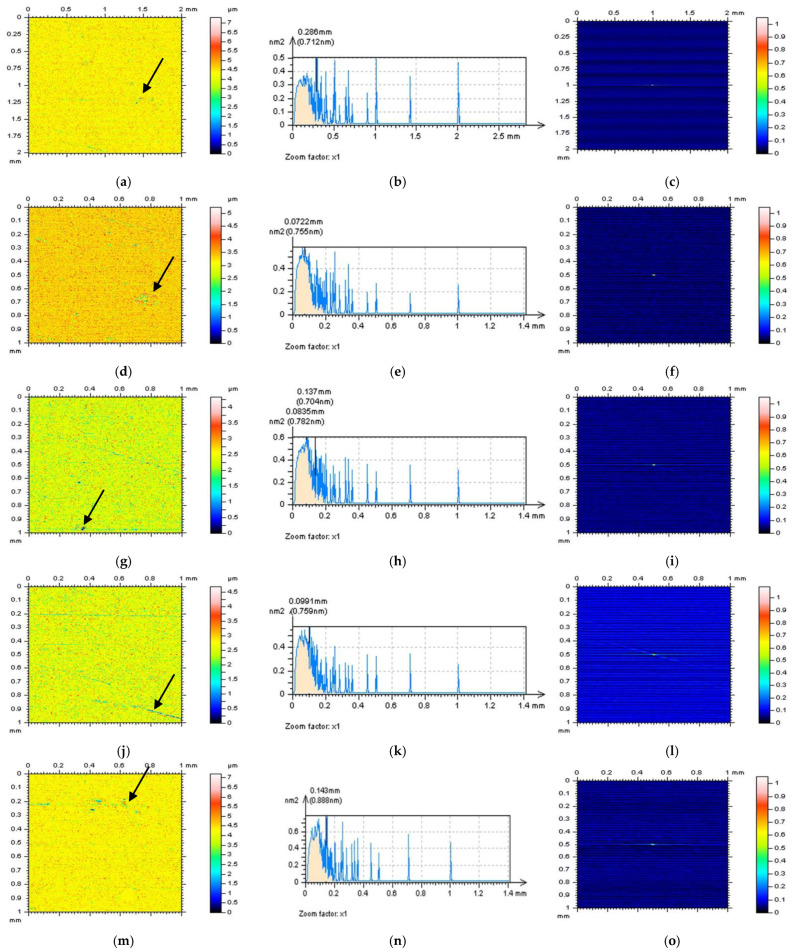
Contour map plots (left column), its 3D PSDs (middle) and ACFs (right column) of NS received by an RGF application (cut-off = 0.015 mm) from the measured surface (**a**–**c**) and surfaces: E (**d**–**f**), F (**g**–**i**), G (**j**–**l**), and H (**m**–**o**) extracted from a turned surface. Description of A–D surfaces was presented in the previous figure, Figure 5. Arrows present unwanted features.

**Figure 7:**
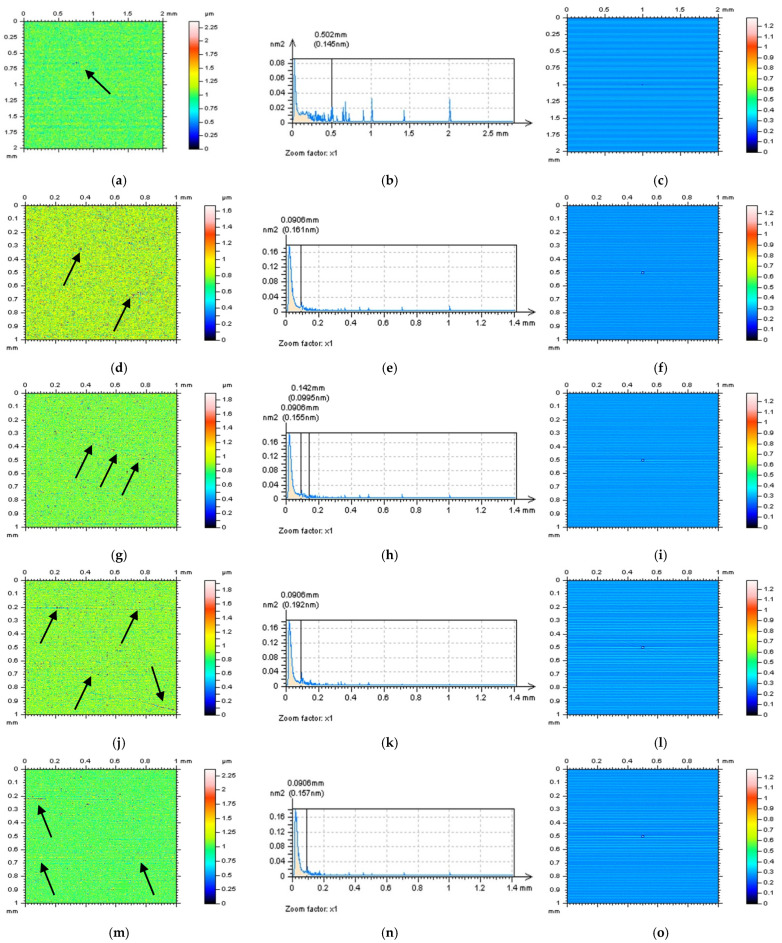
Contour map plots (left column), its 3D PSDs (middle) and ACFs (right column) of NS received by an SF filter application (cut-off = 0.015 mm) from the measured surface (**a**–**c**) and surfaces: E (**d**–**f**), F (**g**–**i**), G (**j**–**l**), and H (**m**–**o**) extracted from a turned topography. The non-noise features were indicated by arrows.

**Figure 8:**
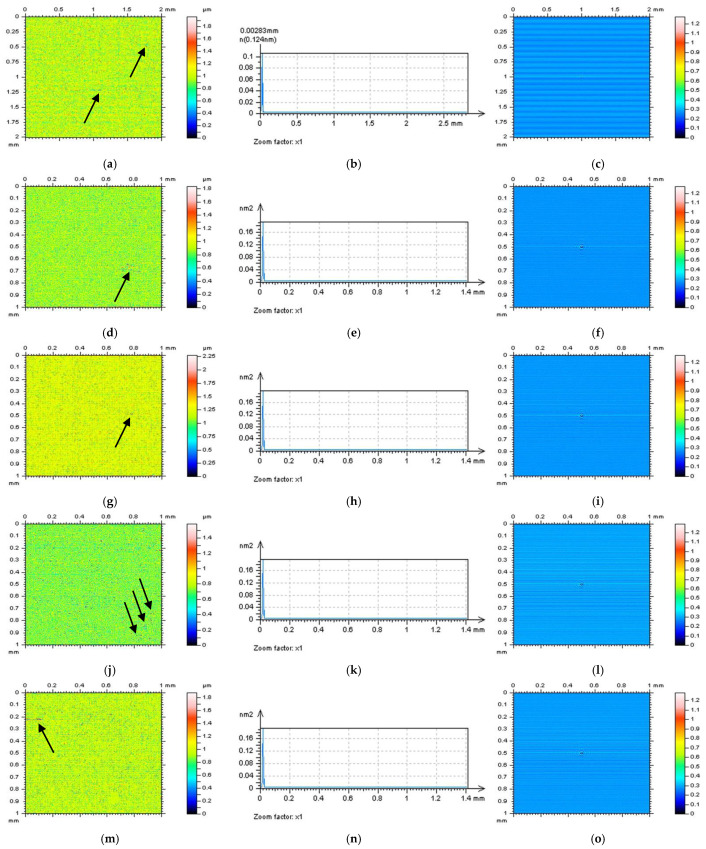
Contour map plots (left column), its 3D PSDs (middle) and ACFs (right column) of NS received by application of the FFTF method (cut-off = 0.015 mm) from the measured surface (**a**–**c**) and surfaces: E (**d**–**f**), F (**g**–**i**), G (**j**–**l**), and H (**m**–**o**) extracted from a turned topography. The features which are not classified as the noise (like scratches, dimples, wholes), were presented by arrows.

In Figure 6, NSs defined by an RGF (cut-off = 0.015 mm) for the whole measured surface (a) and each of the extracted surfaces are presented: E(d), F(g), G(j), and H(m). For each of the studied surfaces, the NSs contain features that cannot be defined (in a non-high-frequency domain). Even the PSDs (middle column) of the NSs indicated the presence of high-frequency domains (Figure 7), the occurrence of some unacceptable features (it was indicated by the arrows), such as dimples and scratches for measured surfaces (a), holes for surface E (d), and scratches for surfaces F (g), G (j), and H (m), makes analysed method inappropriate. When another method (filter) was used (e.g., FFTF), additional features were also defined on the NSs (Figure 8). However, from all of the methods applied (GF, RGF, SF, and FFTF), the most encouraging results when studying the isometric view of the NSs were received when an FFTF was used. This method can be classified as the most suitable for the detection of high-frequency measurement errors and has been proposed in previous studies [36].

### 3.2. Selection of Cut-Off Value with a Feature-Based Technique

When selecting a method (filter) for the suppression of high-frequency measurement noise, the value of the cut-off must also be preferred. Its validity can significantly influence the distortion of results obtained and values of ISO 25178 surface texture parameters. It was found in previous studies that properly defined NSs can be classified as presenting an appropriate method for high-frequency noise reduction. When selecting the cut-off value, the NS should contain only high-frequency components and not contain other features representing different frequencies [57]. Holes, dimples, scratches, or other non-high-frequency-noise features (shortly non-noise features), represent frequencies in a different domain than a high-frequency spectrum. It was also previously indicated that the occurrence of non-noise features caused NS was not isotropic [16]. Therefore, both properties require the NS to be in the high-frequency domain and isotropic. For that performance, it is required to increase the value of the cut-off until non-noise features occur.

**Figure 9:**
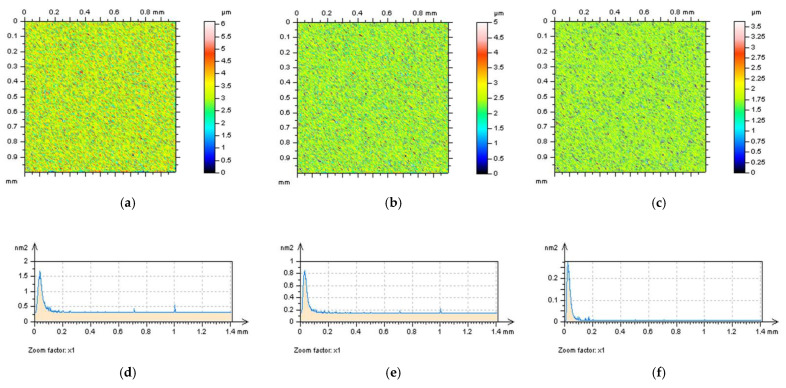

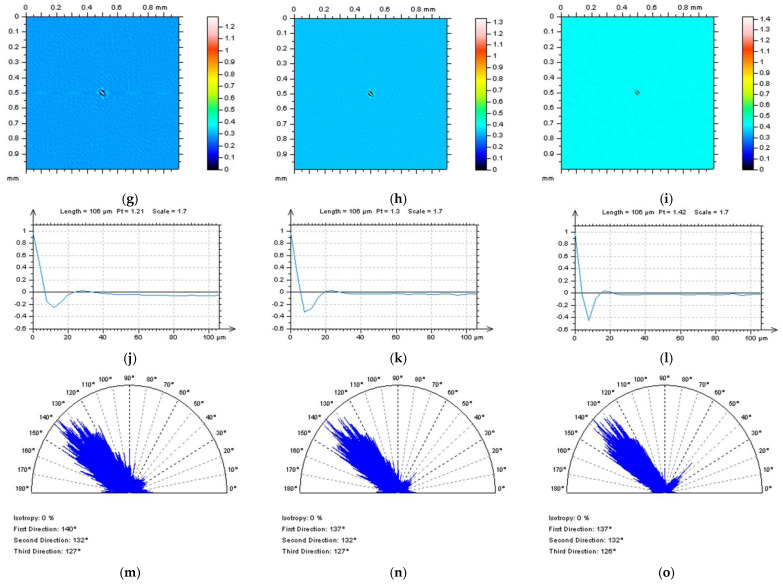
Contour map plots (**a**–**c**), its 3D PSDs (**d**−**f**), 3D (**g**−**i**), and 2D (**j**−**l**) ACFs and TDs (**m**−**o**) received from NSs defined by an application of SF with cut-off equal to: 0.035 mm (left column), 0.025 mm (middle), and 0.015 mm (right column), obtained for surface D.

In Figure 9, the NSs defined by an SF for surface D (not containing features in primary, raw measured data) do not contain any non-noise features; nevertheless, preliminary data also did not contain features, so it is difficult to expect features on the NS. Moreover, the NS contain only high-frequency components when analysing the PSD graph. For that matter, it looks like 0.035 mm might be suitable for high-frequency measurement noise reduction. However, the received NS is not isotropic when studying the TD graph. This indicates that even though it is not visible in the isometric view and on the PSD graph, the NS can contain some non-noise features. Furthermore, this trend was found for each of the cut-off values, 0.035 mm, 0.025 mm, and 0.025 mm. From that perspective, the SF with a small-scale cut-off value (0.015 mm) is not suitable for the reduction of the high-frequency errors from the results of surface topography measurement of turned surfaces.

**Figure 10:**
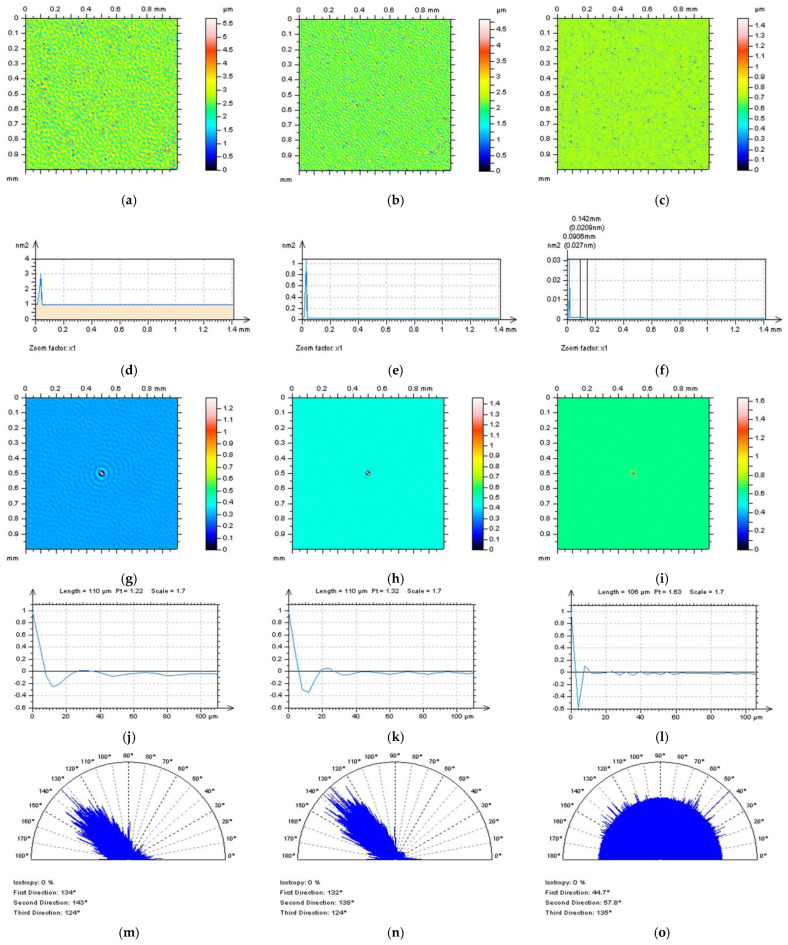
Contour map plots (**a**−**c**), its 3D PSDs (**d**−**f**), 3D (**g**−**i**), and 2D (**j**−**l**) ACFs and, correspondingly, TDs (**m**−**o**) of NSs received by an application of FFTF with cut-off equal to: 0.035 mm (left column), 0.025 mm (middle), and 0.015 mm (right column), obtained for surface B.

Validation of the method (filter) should be improved for surfaces containing certain features, such as surface B. In this case, the surface contains one valley feature. It is suggested to observe the area on the NS where this feature is located. In Figure 10, it NSs can be observed when an FFTF is applied for various bandwidth values, e.g., 0.035 mm, 0.025 mm, and 0.015 mm. For all three values, the isometric view of the NSs and PSD graphs do not indicate non-noise features, especially in the location where the feature occurred. However, both NSs, defined at 0.035 mm and 0.025 mm bandwidth values, were not isotropic. This indicates that some features of the surface have their own directions. On the other hand, the direction can indicate feature occurrence; therefore, the TD graph can indicate falsely estimated NSs (filter or its cut-off value) even though non-noise features are recognisable.

Some improvement in the observation received can also be found when analysing PSD. Even though each of the three cut-off values (Figure 10d–f) represent proper PSD justifications, PSD from the NS obtained while 0.015 mm FFTF bandwidth was applied confirmed the selection of an appropriate cut-off value (Figure 10f). It can also be observed that the smallest value provided the most high-frequency components on the NS.

Another advance was also achieved received when studying the ACF shape. It was found in previous studies that for surfaces containing high-frequency errors, the shape of the centre part of ACF was different than for surfaces without that type of noise. Usually, the centre part (maximum value) of ACF increases more rapidly when high-frequency errors are included in the data. These are especially visible when a 2D (profile) ACF is considered. This can be achieved by extracting the centre part of 3D ACF or by providing ACF for profile (2D) data. Both methods can provide similar results; nevertheless, when analysing areal (3D) surface topography with areal surface topography parameters (ISO 25178), it is suggested to extract relevant (centre in this case) profiles to study this property. In conclusion, a multithreaded method, including analysis of the isometric view, PSD, ACF, and TD of NS, is required for the validation of the filtering method and its bandwidth characteristics (cut-off values) for high-frequency noise detection and reduction. 

## 4. The Outlook

Despite all of the detailed analysis provided, some limitations of the present study must be discussed in future work.

1. Feature-based characterisation for the suppression of high-frequency measurement noise cannot be validated for isotropic surfaces where the properties of NSs (such as defining features and texture direction) are difficult to be determined. In this case, it is difficult to find any features on the NSs even though a relatively huge (e.g., 0.035 mm) filter bandwidth is proposed. Increasing the bandwidth would remove essential features in the roughness evaluation process.
2. The proposed technique was not convincing when the surface contained relatively shallow features, such as holes, dimples, and oil pockets. In this case, the detection of non-noise features on the NS can be difficult. However, the parameters (size, depth, width, and density) of features would depend on the surface height (both Sa and Sz values).
3. For isotropic surfaces, the definition of the cut-off value can be even more complicated than increasing the bandwidth value, e.g., from 0.025 mm to 0.35 mm, and may not allow for the detection of unwanted features on the NS. Moreover, the selection of a proper filter would also be difficult. Increasing bandwidth can cause more distortions in surface features, especially wide and deep dimples (valleys). For that matter, analysis of the ACF shape and TD graph may be reasonably required.
4. A feature-based method can be applied to many other types of surface texture where direction can be defined, such as plateau-honed cylinder liner topographies, ground, milled, and laser-textured, with a defined direction of features in general. However, when selecting the algorithm (e.g., filter) and, especially, its bandwidth value, different features with various locations and densities must be characterised. The distortion of results received by digital filtering can arise when certain inappropriate methods (filters) are applied.

## 5. Conclusions

From all of the proposals presented in the paper, the following main issues can be concluded:

1. High-frequency measurement noise can affect the results of surface topography analysis, especially when considering the ISO 25178 parameters. The whole reduction process of this type of error can be divided into two separate but tightly connected processes: detection (definition) and removal (reduction).
2. For the detection of high-frequency errors in surface metrology, it is suggested to use a multithreaded technique, where various analysis methods are applied simultaneously. In the validity of the detection process, many regular techniques available in commercial software are sufficient, e.g., PSD, ACF, and TD techniques. The application of those commonly available methods can improve the detection process significantly.
3. Feature-based techniques can be especially valuable in improving the detection and reduction processes of high-frequency measurement noise that can help better validate NS properties. The definition of NSs with required characteristics can reduce the errors in high-frequency measurement noise reduction.
4. When selecting the bandwidth value of digital filtering to suppress high-frequency measurement errors, all of the techniques, feature-based, PSD, ACF, and TD, must be provided simultaneously. It is suggested to increase the value of the cut-off until the NS contains non-high-frequency-noise features, such as dimples, holes, scratches, etc. When NSs contain unwanted features, the value of the filter bandwidth must be decreased.
5. It was observed that the selection of filter bandwidth depends on the size and density of the features in surface texture. However, the smaller the number of features on the raw measured data detected, the more attention must be paid when selecting the method (filter) and its bandwidth (cut-off) value. The occurrence of the surface features is crucial in the detection and reduction of considered high-frequency measurement errors, so any feature-based technique can be highly advantageous in roughness evaluation and the calculation of ISO 25178 surface topography parameters.

## Data Availability

Data sharing is not applicable to this article.

## Nomenclature

The following abbreviations and surface topography parameters are used in the manuscript:

ACF	autocorrelation function
FFT	fast Fourier transform
FFTF	fast Fourier transform filter
GF	regular Gaussian regression filter
NS	noise surface
PSD	power spectral density
RGF	robust Gaussian regression filter
SF	regular isotropic spline filter
TD	texture direction (graph)
Sa	arithmetic mean height Sa, µm
Sal	auto-correlation length, mm
Sbi	surface bearing index
Sci	core fluid retention index
Sdq	root mean square gradient
Sdr	developed interfacial areal ratio, %
Sk	core roughness depth, µm
Sku	kurtosis
Smc	inverse areal material ratio
Smr	areal material ratio
Sp	maximum peak height, µm
Spc	arithmetic mean peak curvature, 1/mm
Spd	peak density, 1/mm^2^
Spk	reduced summit height, µm
Sq	root mean square height, µm
Ssk	skewness
Std	texture direction, °
Str	texture parameter
Sv	maximum valley depth, µm
Svi	valley fluid retention index
Svk	reduced valley depth, µm
Sxp	extreme peak height
Sz	the maximum height of surface, µm
